# Natural nutrient subsidies alter demographic rates in a functionally important coral-reef fish

**DOI:** 10.1038/s41598-021-91884-y

**Published:** 2021-06-15

**Authors:** Cassandra E. Benkwitt, Brett M. Taylor, Mark G. Meekan, Nicholas A. J. Graham

**Affiliations:** 1 Lancaster Environment Centre, Lancaster University, Lancaster, UK; 2 Indian Ocean Marine Research Centre, Australian Institute of Marine Science, Crawley, WA Australia; 3 Present Address: University of Guam Marine Laboratory, UOG Station, Mangilao, GU USA

**Keywords:** Ecology, Marine biology, Ichthyology, Ecology, Conservation biology, Ecosystem ecology, Invasive species, Population dynamics, Stable isotope analysis

## Abstract

By improving resource quality, cross-ecosystem nutrient subsidies may boost demographic rates of consumers in recipient ecosystems, which in turn can affect population and community dynamics. However, empirical studies on how nutrient subsidies simultaneously affect multiple demographic rates are lacking, in part because humans have disrupted the majority of these natural flows. Here, we compare the demographics of a sex-changing parrotfish (*Chlorurus sordidus*) between reefs where cross-ecosystem nutrients provided by seabirds are available versus nearby reefs where invasive, predatory rats have removed seabird populations. For this functionally important species, we found evidence for a trade-off between investing in growth and fecundity, with parrotfish around rat-free islands with many seabirds exhibiting 35% faster growth, but 21% lower size-based fecundity, than those around rat-infested islands with few seabirds. Although there were no concurrent differences in population-level density or biomass, overall mean body size was 16% larger around rat-free islands. Because the functional significance of parrotfish as grazers and bioeroders increases non-linearly with size, the increased growth rates and body sizes around rat-free islands likely contributes to higher ecosystem function on coral reefs that receive natural nutrient subsidies. More broadly, these results demonstrate additional benefits, and potential trade-offs, of restoring natural nutrient pathways for recipient ecosystems.

## Introduction

Energy acquisition and allocation strategies vary across populations due to a variety of biotic and environmental factors, resulting in intraspecific variation in demographic rates over a range of spatial scales^1–6^. Understanding the drivers and consequences of these differences in individual demographic rates provides a key link to population and community dynamics^7,8^, and can be used to identify areas of high conservation priority, for example hotspots of enhanced growth and reproduction^9^.

Many ecosystems receive allochthonous nutrient inputs via the movement of mobile consumers, which in turn can boost the availability and quality of food resources^10,11^. With higher resource acquisition, trade-offs in energy allocation can be reduced, thus boosting multiple demographic rates^12–14^. Humans are disrupting the flow of natural nutrient subsidies, however, through activities such as overharvesting, habitat degradation, and the introduction of exotic predators^11^. For example, seabirds transport large quantities of nutrients from offshore pelagic food webs to terrestrial and coastal systems by feeding in the open ocean and returning to islands where they roost and breed^10,15^. However, introduced rats feed on nesting seabirds, chicks, and eggs, and have consequently decimated seabird populations in 90% of the world’s island chains^16^. Despite the importance of cross-system nutrient subsidies^10,11^, it is unclear how their loss influences multiple demographic rates in consumers, and how this in turn influences population dynamics and ecosystem functioning.

On nearshore coral reefs, the importance of breeding seabirds in providing natural nitrogen inputs, and thus bolstering fish biomass, productivity, and ecosystem functioning, has been recently recognized^17,18^. This includes evidence that a small species of herbivorous damselfish grows faster around islands with abundant seabird populations compared to islands with invasive rats and thus few seabirds^17^. These damselfish consume turf algae within small territories, and elevated levels of seabird-derived nutrients are present in both turf algae and damselfish tissues, providing a causative pathway between seabird presence and enhanced fish growth rates^17^. However, it is unknown whether seabird-derived nutrients affect the growth of larger species that perform key functions on coral reefs, such as parrotfishes. Parrotfishes primarily consume microscopic autotrophs, namely cyanobacteria on and within the reef matrix, which they access with their powerful beak-like jaws^19,20^. In doing so, they perform several ecosystem functions, including scraping the substrate clear of algae and bioeroding dead reef substrate, thus producing and transporting sediment^21^. Importantly, the functional significance of parrotfishes scales non-linearly with body size^22–25^, providing a direct link between individual growth rates and ecosystem function.

Whether seabird-derived nutrients affect the reproductive investment of coral-reef fish is currently unknown. Like most benthic marine organisms, local populations of coral-reef fish are linked via a dispersive larval phase, meaning there could be far-reaching effects of seabird-derived nutrients beyond the spatial scale of the subsidy itself. For example, if fish reproductive output is greater around islands with seabirds than islands with invasive rats, then these fish could serve as source populations for other reefs, much as populations within marine protected areas seed adjacent reefs^26^. Thus, studying the simultaneous effects of nutrient subsidies on growth and reproduction of reef fishes will not only determine their influence on life-history trade-offs, but could also inform our understanding of the spatial extent of nutrient effects.

Here, we test the influence of nutrient subsidies from seabirds on the demographic rates of the widespread and functionally-important parrotfish *Chlorurus sordidus.* Specifically, we test the hypothesis that seabirds increase the growth and reproductive investment of *C. sordidus* by comparing parrotfish around rat-free islands with high densities of breeding seabirds to those around nearby rat-infested islands with few seabirds. Using visual survey data, we then determine whether changes in these demographic rates coincide with population-level attributes (density, biomass, and size distribution) on reefs adjacent to rat-free versus rat-infested islands. We expect increased growth rates to enhance overall parrotfish biomass and size distributions, whereas density could be a driver of demographic differences due to density-dependent competition or predation. Finally, we examine other potential bottom-up and top-down drivers of demographic variation, focusing on nitrogen (a key nutrient) in parrotfish tissue and piscivorous fish populations.

## Methods

### Study sites, collections, and surveys

This study was conducted in the remote northern atolls of the Chagos Archipelago, Indian Ocean. Due to its status as a large (550,000 km^2^) marine protected area and its relative isolation, there is no fishing allowed or other direct human influences in the study atolls^27–29^. The Chagos Archipelago is also home to eighteen species of breeding seabirds, many of which are present in globally-significant numbers, leading to the designation of several Important Bird and Biodiversity Areas (IBAs) within the archipelago^30^. However, the distribution of seabirds throughout the archipelago is patchy due to the presence of black rats (*Rattus rattus*) on some islands, which were introduced several hundred years ago^31^. As a result of rat predation, seabird density is an estimated 760 times greater on rat-free islands than rat-infested islands, which in turn leads to ~ 251 times greater nitrogen input by seabirds to these islands and enhanced seabird-derived nutrients in sponges, algae, and herbivorous damselfish on adjacent coral reefs^17^. Seabird-derived nutrients likely enter the marine environment throughout the year, with both high rainfall and breeding seabirds occurring in every month^30,32^. In contrast to seabirds, which import nutrients from highly productive, offshore, open ocean areas^17^, invasive rats recycle nutrients that are already present on the islands. Thus, seabirds, but not rats, add substantial amounts of allochthonous nitrogen to islands and nearshore reefs.

For the specific islands used in this study, we obtained data on the densities of breeding seabirds from^30^, sea surface temperature from NOAA, wave exposure from^33,34^, and net primary productivity from^34^ (see Supplementary Table S1 for details). The presence versus absence of invasive rats, and thus seabirds, was the greatest difference among islands (*Results*, Supplementary Tables S1 and S2, Supplementary Figs. S1 and S2). Sea surface temperature was similar across all of the islands, and although there was some atoll-level variation in wave exposure and net primary productivity, there were no consistent differences between rat-free versus rat-infested islands (Supplementary Tables S1 and S2, Supplementary Figs. S1 and S2). Rat-free islands tended to be smaller than rat-infested islands, but island size is not expected to be a strong driver of parrotfish demography, especially across the range of island sizes investigated here (all < 244 ha). Therefore, we used the large differences in seabird-derived nutrient subsidies between islands with and without rats to test how nutrient inputs from seabirds affect the demography of parrotfish.

Bullethead parrotfish (*C. sordidus*) provide an ideal model with which to study the influence of nutrient subsidies on growth and reproduction for several reasons. Like most wrasses and parrotfishes, *C. sordidus* is a protogynous hermaphrodite, with distinct colouration patterns for each phase (immature, female [initial phase], and male [terminal phase], with rare occurrence of initial phase primary males^35^). Although the spawning frequency of *C. sordidus* is unknown, its sister species in the Pacific (*C. spilurus*, formerly *C. sordidus*) has an extended spawning season and can spawn throughout the lunar cycle^36^. Combined, these characteristics increased our likelihood of capturing spawning-capable females. Their high dispersal ability as larvae means there is little genetic separation at small to medium scales^37^, so any observed differences among individuals from different islands are likely to be driven by local differences in environmental conditions (e.g., seabird/rat presence) rather than genetic differences. Adults have relatively small home ranges (maximum linear distance 1.2 km along continuous reefs) and are unlikely to cross sand channels or move among reefs^4,26,38^, so we can assume individuals spend most of their time near where they were caught.

We haphazardly collected *C. sordidus* displaying initial phase colouration from shallow coral reefs on the lagoonal sides of 8 islands (4 rat-infested and 4 rat-free) from 4 to 16 March 2019 (n = 7–17 individuals per island, Supplementary Fig. S1). Although it is likely that *C. sordidus* spawns throughout the lunar cycle, to control for any temporal variation in reproductive investment we paired collections of fish from both rat-free and rat-infested islands within each of 3 atolls. Thus, all fish from Peros Banhos atoll were collected within 5 days (2 rat-free and 2 rat-infested islands, within 3 days of the new moon), fish from Salomon atoll were collected within 1 day (1 rat-free and 1 rat-infested island, 4 days after the new moon), and fish from the Great Chagos Bank were collected within 3 days (1 rat-free and 1 rat-infested island, 8–10 days after the new moon). Available information from this species and sister species *C. spilurus* suggests a high frequency of spawning activity throughout the lunar period, resulting in a temporally consistent gonad state (B. Taylor, unpublished data)^36^. All fish were caught from the reef crest and shallow slope within approximately 250 m from shore, where the signals from island-based nutrients are strongest^17^. Importantly, the habitats where we collected fish were similar across all islands; they were all sheltered, lagoonal reefs. All sampling was done in accordance with institutional and local guidelines, with ethical approval granted by the Lancaster University Animal Welfare and Ethical Review Body (AWERB permit number A100143).

For all fish, we measured total length (TL, cm) and wet weight (WW, kg) (size range = 14.0 to 26.8 cm TL and 0.05 to 0.39 kg WW). A sample of white dorsal muscle was taken and dried in an oven at 60˚C for 48 h for stable isotope analysis. Otoliths were removed and stored dry for aging analysis. Sex was determined by macroscopic examination of the gonads, and for fish identified as female, reproductive stage was classified as in^39^, and gonads were removed for further analysis. Gonads were fixed in 10% neutral-buffered formalin and then transferred to 70% ethanol for storage before histological analysis. We collected and identified 98 spawning capable (i.e., mature or ripe) females, 3 immature individuals, 2 transitional individuals, 1 initial phase male, and 11 regenerating (i.e., resting or inactive but mature) females.

To determine the population-level characteristics of parrotfish, we conducted underwater visual censuses around 10 islands (5 rat-infested and 5 rat-free) in May 2018 and 1 rat-infested island in March 2019, with five of these survey sites matching the collection sites (Supplementary Fig. S1). We conducted surveys and collections as close in time and space as possible, with surveys always conducted prior to the collections. Although logistical constraints prevented conducting all surveys and collections in the same year, several lines of evidence suggest that it is appropriate to interpret the results from the collections and surveys in tandem. First, herbivore populations have remained stable on these reefs from 2015 to 2018^40^, and no major disturbances occurred between the majority of surveys (2018) and the majority of collections (2019). In addition, all of the parrotfish collected were at least 1 year of age (see *Results*), meaning that they were inhabiting the reefs at the time of the population surveys.

Fish were counted along four replicate 30-m transects, spaced 10 m apart, near the reef crest on the lagoon-side of each island (within approximately 325 m from shore), which matches the locations of the fish collections. Along each transect, one observer (NAJG) counted and estimated the size (TL, to the nearest cm) of all diurnal non-cryptic fishes > 8 cm TL, including *C. sordidus*, within a 5-m wide belt. Biomass was calculated using published length–weight relationships^41^. The surveys provide an aggregate picture of the population density, biomass, and size structure of *C. sordidus* on these reefs, as the colouration (immature, initial phase, or terminal phase) of individuals was not recorded. From the same surveys, we also gathered data on other factors that could influence parrotfish demography. Specifically, we estimated the density, biomass, and size structure of piscivorous fishes using the methods as for *C. sordidus*. We estimated hard coral cover using the point-intercept method, as coral cover is negatively related to parrotfish food availability^20,42^. Finally, we determined structural complexity using a standard scale ranging from 0 (no relief) to 5 (exceptionally complex relief)^43,44^. Data on piscivore biomass, coral cover, and structural complexity is previously published in^40^.

### Laboratory analyses

To determine nutrient subsidy signals in the parrotfish, we quantified total nitrogen (%) and ratio of isotopic δ^15^N in dorsal muscle tissue from each parrotfish. Elevated δ^15^N values reflect seabird-derived nitrogen in nearshore tropical marine food chains, as seabird guano is high in δ^15^N in part because they feed on high trophic levels in the open ocean^17,45,46^. Analyses were conducted at the stable isotope facility at Lancaster University (Lancaster, UK). All samples were combusted using an Elementar Vario Micro Cube Elemental Analyser and analysed using an Isoprime 100 Isotope Ratio Mass Spectrometer, with international standards IAEA 600 and USGS 41. Accuracy based on internal standards was within 0.2 permil standard deviation. Selected samples were run in duplicate to further ensure accuracy of readings.

Parrotfish otoliths were cross-sectioned to determine age (in years) from annual bands for all individuals. One randomly selected otolith from each pair was mounted to the edge of a glass slide using thermoplastic glue (Crystalbond 509) with the otolith core situated directly inside the slide edge. The otolith material was sanded away to the slide edge using a 1200-grit diamond lap on a lapping machine with constant water flow. The slide was heated (200 °C) and remounted with the newly sanded surface placed flat against the slide, and then the remaining bulk of otolith material was sanded away until a thin transverse cross-section (150 µm) remained. Annuli, denoted by alternating opaque and translucent growth bands, were counted using a stereo-microscope three separate times by the same observer, who was blinded to any characteristics of the fish including collection site, treatment, size, and previous otolith readings. Fish age (in years) was assigned when two or more counts agreed, which was achieved for all specimens.

Gonads were weighed to the nearest 0.1 g. We were unable to weigh fresh gonads due to the inaccuracy of balances on the research vessel, so gonads were weighed on shore after being fixed in formalin. Formalin-fixed gonad weight is scalable to fresh weight^47^, and any effect of formalin fixation should be equal across all samples. To confirm macroscopic staging, one lobe from each gonad was used for histological analysis at the Centre for Environment Fisheries and Aquaculture Science (CEFAS, Weymouth, UK). Samples were processed in a Thermo Excelsior AS vacuum infiltration processor, using standard histology protocols. Tissues were embedded in paraffin wax, sectioned (3 μm) using a rotary microtome and stained with haematoxylin and eosin using a Thermo Scientific Varistain Gemini ES automated stainer. Slides were analysed using a Nikon Eclipse E800 microscope and micrographs were captured using the Nikon NIS Elements BR image analysis software.

A total of 96 females were confirmed as being spawning capable, so were used in all subsequent analyses. All ovaries contained vitellogenic oocytes, but not hydrated oocytes or post-ovulatory follicles. Thus, although all fish were spawning capable, they were not actively spawning nor had they recently spawned during this cycle^39^. However, given the capability for daily spawning and rapid egg development in parrotfishes, these fish could become active spawners within days. We were unable to count and measure oocytes to determine batch fecundity and mean egg size due to the lack of hydrated oocytes, as estimates from earlier egg stages are unlikely to provide accurate estimates for these metrics. However, especially because all fish were in the exact same reproductive stage, gonadosomatic index (GSI, gonad weight/body weight × 100) provides a reasonable measure of relative reproductive investment, and GSI has been used as an indicator of reproductive investment in previous demographic studies of coral-reef fishes, including of the sister species *C. spilurus*^4,6,48,49^.

### Statistical analyses

To determine the effects of seabird subsidies on parrotfish demography, we used a series of multi-level Bayesian models (additional details on model specifications provided in Supplementary Methods). Most of our response variables were modelled as a function of rat status (rat-infested with few seabirds versus rat-free with abundant seabirds) with the intercept allowed to vary by the atoll within which each island was located, which helped account for atoll-level environmental differences (Supplementary Table S1). This model framework was used for percent nitrogen, δ^15^N, GSI, density, biomass, and size distribution of *C. sordidus*, as well as for island, reef, and environmental characteristics that may help explain any observed differences in *C. sordidus* demography (Supplementary Table S1). Full descriptions of these models are provided in the supplement, while deviations from this framework are described in detail below.

Models for GSI included fish length as an additional covariate. To ensure that the estimated effect of rat status on GSI was robust to any temporal variation in reproductive investment (see *Study sites, collections, and surveys*) and observed differences in size-at-age around rat-free and rat-infested islands (see *Results*), we ran several model variations and report the results from these models in the supplement. Specifically, we included collection day as an additional explanatory variable to account for potential temporal variation, but there was no evidence that this improved model fit. We also modelled GSI as a function of age instead of length. The estimated difference in GSI between rat-free and rat-infested islands was nearly identical in all models (Supplementary Methods).

Density and biomass of *C. sordidus* from underwater visual surveys were modelled following hurdle gamma distributions with log links because the data were zero-inflated, and size was modelled following an exponentially-modified normal distribution, which enabled us to determine the effect of rat presence on both mean size and the skewness of the size distribution. We ran all models both for all 11 surveyed islands, and for only the 5 surveyed islands which exactly matched collection islands. The results were qualitatively similar, so we present the models with all islands, and thus a larger sample size.

Following^17^, *C. sordidus* growth was modelled using the von Bertalanffy growth function as:

$$L_{t} = L_{\infty } - \left( {L_{\infty } - L_{0} } \right)e^{{ - \left( {k + k_{b} } \right)t}}$$

where $L_{t}$ is the observed length at age *t*, $L_{\infty }$ is the estimated asymptotic length, $L_{0}$ is the theoretical length at age 0, and *k* is the estimated growth coefficient towards $L_{\infty }$, with an additional term $k_{b}$ providing an offset to compare *k* around rat-free versus rat-infested islands. By including the term $k_{b}$ we allowed *k*, but not $L_{0}$ and $L_{\infty }$, to vary between rat-free and rat-infested islands. There was good reason to constrain $L_{0}$ and $L_{\infty }$ in this way, as $k_{b}$ can then be interpreted as the difference in growth rate between rat-free and rat-infested islands, which is more easily interpretable and biologically-relevant than the difference in growth towards maximum size. Thus, $k_{b}$ = 0 if there is no difference in growth between rat-free and rat-infested islands, $k_{b}$ > 0 if growth is faster around rat-free islands, and $k_{b}$ < 0 if growth is slower around rat-free islands. This parameterization was previously used to compare growth of herbivorous damselfish between many of the same rat-free and rat-infested islands used in our study^17^, so using the same model also enables us to directly compare our results for parrotfish to those of damselfish. However, to ensure any observed differences in growth were not simply due to this parametrization, we ran an additional model where we allowed both $L_{\infty }$ and *k* to vary by rat status. We found the estimated growth curves and difference in *k* between rat-free and rat-infested models were similar in the two models, and there was no evidence that $L_{\infty }$ differed between rat-free and rat-infested islands even when it was allowed to vary (Supplementary Methods). Furthermore, the model fit was best when only *k* was allowed to vary by rat status, compared to the model in which both $L_{\infty }$ and *k* were allowed to vary, as well as a model in which neither were allowed to vary (Supplementary Methods).

To further explore differences in growth between rat-free and rat-infested islands, we also modelled length-at-age using a reparameterization of the VBGF in which lengths are estimated for three ages: *lphi*, *lpsi*, and *lchi* (where *lphi* < *lpsi* and *lchi* is the mean of *lphi* and *lpsi)*^50^. We set *lphi* = 2, *lpsi* = 6, and *lchi* = 4, with *lphi* and *lpsi* chosen to maximize the range of ages for which we had sufficient data from both rat-free and rat-infested islands. Finally, we compared the maximum age and maximum length of collected parrotfish between rat-free and rat-infested islands by comparing the island-level means of the upper quartile of ages and lengths^51^. Because we only examined female IP fish, differences in these parameters correspond with differences in age- and size-at-sex change, rather than differences in absolute maximum age and size.

For all models, we used weakly informative priors and selected distributions based on the underlying data in conjunction with model diagnostics (Supplementary Methods)^52^. All models were run for 4 chains, with at least 3,000 iterations and a warm-up of 1,000 iterations. We conducted graphical posterior predictive checks comparing model fits to the data, and evaluated convergence via traceplots and the Gelman-Ruban convergence diagnostic (R-hat)^52^. All analyses were conducted in R and implemented in STAN using the package brms^53,54^. All data and code are publicly available on GitHub (github.com/cbenkwitt/nutrients-fish-demography).

## Results

### Parrotfish growth and fecundity

**Figure 1:**
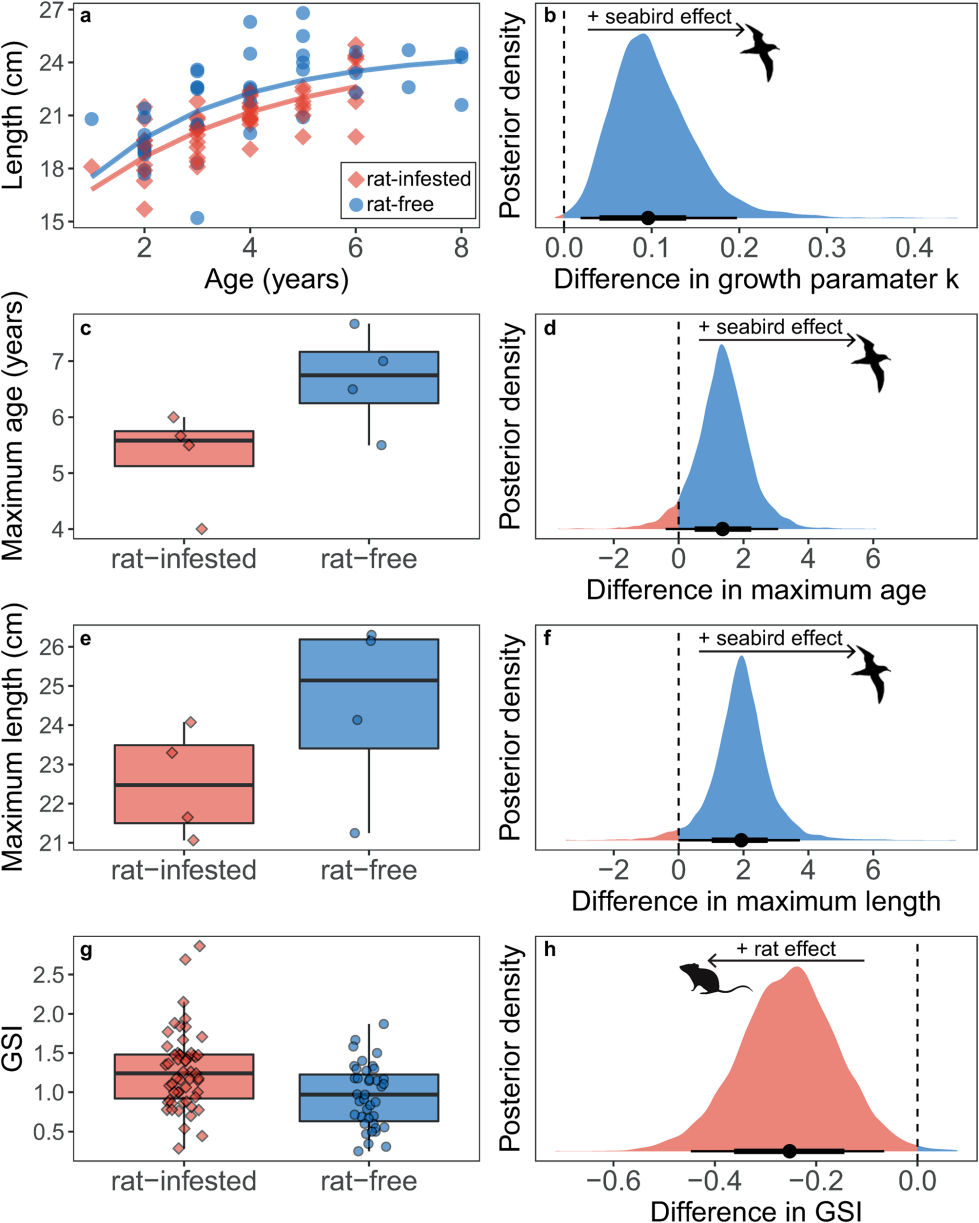
Demographic rates for female *Chlorurus sordidus* around rat-free islands with abundant seabirds (blue, circles) compared to rat-infested islands with few seabirds (red, diamonds). (**a**, **g**) Each point represents measured values from an individual *C. sordidus*, (**c**, **e**) each point represents one island. (**a**) Curves represent fitted estimates from Bayesian models, (**c**, **e**, **g**) box limits represent first and third quantiles (25% and 75% percentiles), middle line represents the median (50% percentile), and whiskers represent smallest and largest observations less than or equal to 1.5 × inter-quartile range. (**b**, **d**, **f**, **h**) Bayesian posterior densities for the effect of seabird presence on the von Bertalanffy growth parameter *k* (**b**), maximum age (**d**), maximum length (**f**), and relative reproductive investment as measured by GSI (**h**). Positive values of the posterior distributions (blue fill) indicate a positive effect of seabird presence on the response, negative values (red fill) indicate a negative effect of seabirds (i.e., positive effect of invasive rat presence). Points represent median highest posterior density estimate and lines represent 75% and 95% highest posterior distribution intervals (HPDI). Rat and seabird silhouettes were obtained from phylopic.org under Public Domain Dedication 1.0 licenses.

Female *C. sordidus* around islands with abundant seabird populations grew an estimated 34.91% faster than those around rat-infested islands with few seabirds (Fig. 1a, b; estimated *k* = 0.38 vs. 0.27; estimated difference in *k* = 0.10, 95% highest posterior density interval [HPDI] = 0.02 to 0.20). This faster growth coincided with parrotfish around rat-free islands being larger for a given age, with the greatest difference occurring at intermediate ages (4 years old) and the difference diminishing by age 6. Parrotfish around rat-free islands were an estimated 0.65 cm, 2.18 cm, and 0.24 cm larger than those around rat-infested islands at ages 2, 4, and 6, respectively (Supplemental Fig. S3; 95% HPDI for difference in length at age 2 =  − 0.33 to 1.61, length at age 4 = 1.18 to 3.42, length at age 6 =  − 1.44 to 1.87). Female parrotfish around rat-free islands also reached older maximum ages (estimated maximum age = 6.64 vs. 5.30 years old, estimated difference = 1.36, 95% HPDI =  − 0.40 to 3.07) and larger absolute maximum sizes (i.e., not maximum size-at-age) (Fig. 1c–f; estimated maximum length = 24.02 vs. 22.10 cm, estimated difference = 1.93, 95% HPDI = 0.00 to 3.74).

By contrast, for a given length, the gonadosomatic index (GSI) of parrotfish around rat-free islands was estimated to be 21.04% lower than the GSI of parrotfish around rat-infested islands (Fig. 1g, h; estimated GSI = 0.94 vs. 1.20; estimated difference =  − 0.25, 95% HPDI =  − 0.45 to − 0.07). There was strong evidence that individual growth rates and GSI were negatively correlated (correlation =  − 0.26, 95% CI =  − 0.41 to − 0.11).

### Parrotfish populations

**Figure 2:**
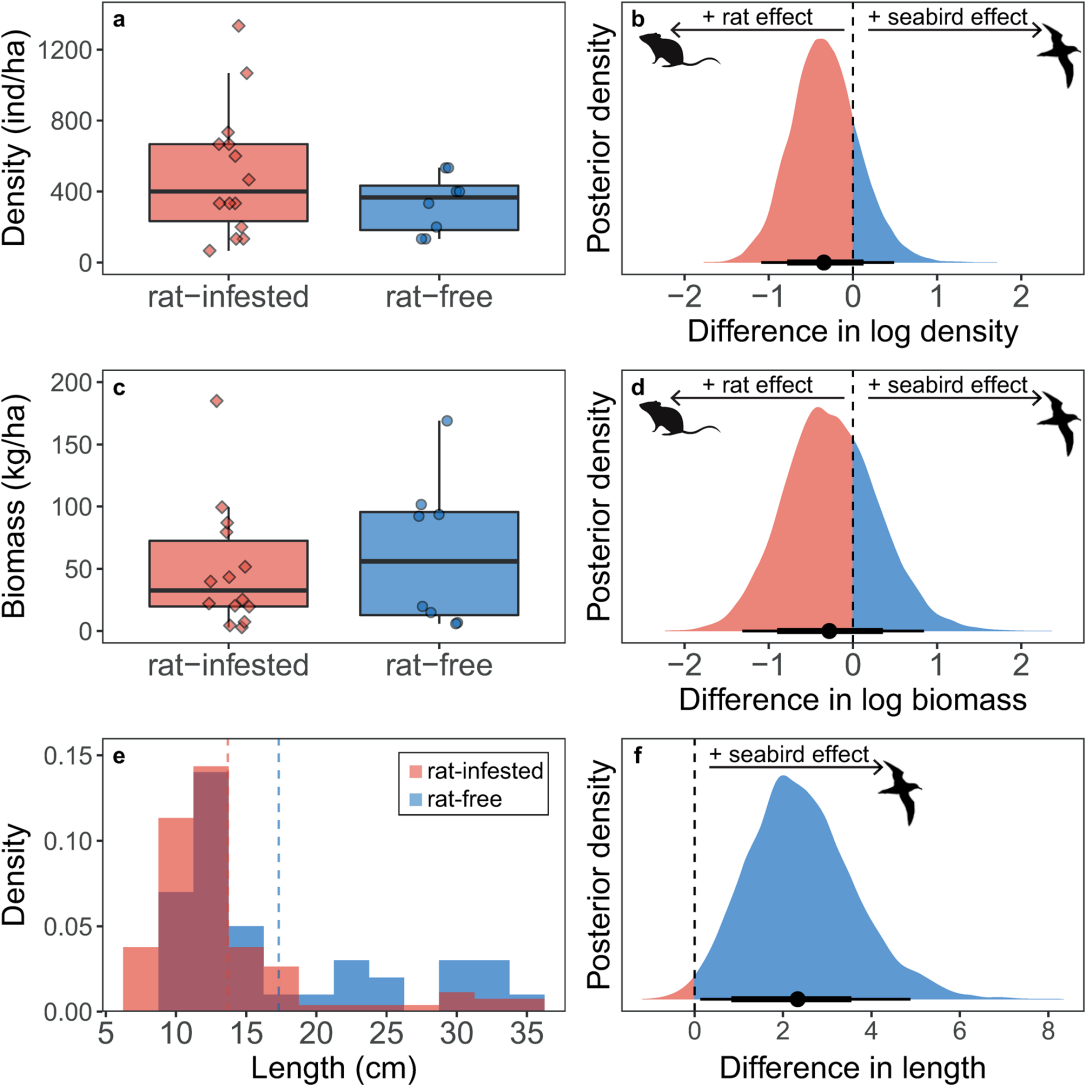
Density, biomass, and size frequency distribution of all *Chlorurus sordidus* around rat-free islands with abundant seabirds (blue, circles) compared to rat-infested islands with few seabirds (red, diamonds). (**a**, **c**) Box limits represent first and third quantiles (25% and 75% percentiles), middle line represents the median (50% percentile), and whiskers represent smallest and largest observations less than or equal to 1.5 × inter-quartile range. Each point represents one transect. Transects on which no *C. sordidus* were observed were excluded from the boxplots (n = 10 around rat-infested islands, n = 12 around rat-free islands) to correspond to estimates from non-zero components of hurdle gamma models presented in (**b**, **d**). (**e**) Size frequency distribution of *C. sordidus* in 2.5 cm size bins. (**b**, **d**, **f**) Bayesian posterior densities for the effect of seabird presence on corresponding responses in (**a**, **c**, **e**). Positive values of the posterior distributions (blue fill) indicate a positive effect of seabird presence on the response, negative values (red fill) indicate a negative effect of seabirds (i.e., positive effect of invasive rat presence). Points represent median highest posterior density estimate and lines represent 75% and 95% highest posterior distribution intervals (HPDI). Rat and seabird silhouettes were obtained from phylopic.org under Public Domain Dedication 1.0 licenses.

Scaling up from individuals to populations, the density and biomass of *C. sordidus* were similar around rat-free versus rat-infested islands (Fig. 2a–d; density back-transformed estimate = 0.70, 95% HPDI = 0.28 to 1.45; biomass back-transformed estimate = 0.78, 95% HPDI = 0.18 to 2.01). However, the overall size distribution of *C. sordidus* was skewed toward larger individuals around islands with abundant seabird populations (estimated difference in skew parameter alpha = 0.46, 95% HPDI = 0.11 to 0.83), and mean length was an estimated 2.33 cm (16.31%) larger around islands with seabirds (Fig. 2e, f; 95% HPDI = 0.13 to 4.88).

### Potential drivers of demographic differences

**Figure 3:**
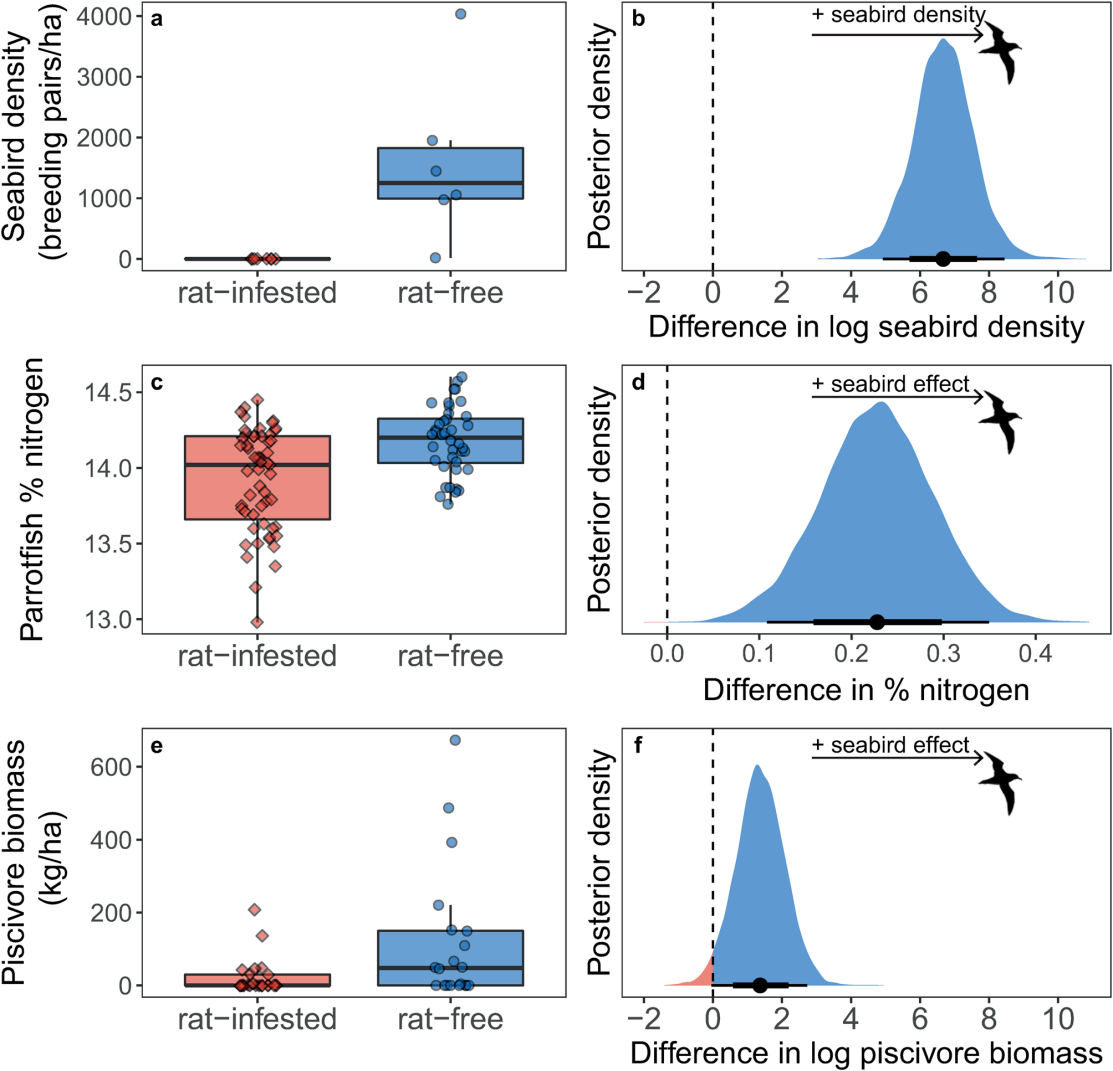
Potential drivers of demographic differences in *Chlorurus sordidus* around rat-free islands with abundant seabirds (blue, circles) compared to rat-infested islands with few seabirds (red, diamonds). (**a**, **c**, **e**) Box limits represent first and third quantiles (25% and 75% percentiles), middle line represents the median (50% percentile), and whiskers represent smallest and largest observations less than or equal to 1.5 × inter-quartile range. Each point represents (**a**) an island, (**b**) measured values from an individual *C. sordidus*, or (**c**) a transect. (**b**, **d**, **f**) Bayesian posterior densities for the effect of seabird presence on the corresponding responses in (**a**, **c**, **e**). Positive values of the posterior distributions (blue fill) indicate a positive effect of seabird presence on the response, negative values (red fill) indicate a negative effect of seabirds (i.e., positive effect of invasive rat presence). Points represent median highest posterior density estimate and lines represent 75% and 95% highest posterior distribution intervals (HPDI). Rat and seabird silhouettes were obtained from phylopic.org under Public Domain Dedication 1.0 licenses.

By far the greatest difference between rat-free and rat-infested islands was the density of breeding seabirds. Seabird density was an estimated 788.52 times higher on rat-free versus rat-infested islands, with similar trends regardless of whether only islands where parrotfish were collected and/or only islands where surveys were conducted were included (Fig. 3a, b, Supplementary Tables S1 and S2, Supplementary Figs. S1 and S2; 95% HPDI = 30.70 to 3307.33).

At an individual-level, *C. sordidus* around islands with abundant seabird populations (rat-free islands) had higher percent nitrogen in their muscle than *C. sordidus* around islands with few seabirds (rat-infested) (estimated difference = 0.23, 95% HPDI = 0.11 to 0.35) (Fig. 3c, d). However, there was no evidence for elevated δ^15^N in parrotfish around rat-free islands (estimated difference = 0.02, 95% HPDI =  − 0.16 to 0.19) (Supplementary Fig. S4).

Piscivore biomass was also greater around rat-free islands by a factor of 3.93 (95% HPDI = 0.42 to 12.73; Fig. 3e, f), which was driven by a combination of both greater densities and larger sizes around rat-free versus rat-infested islands (Supplementary Fig. S5; estimated difference in density = 2.70 times, 95% HPDI 0.16 to 9.85, estimated difference in mean size = 7.70 cm, 95% HPDI = 1.91 to 13.57). By contrast, other external factors that can influence fish demography, including coral cover, structural complexity, sea temperature, net primary productivity, and wave exposure, were all similar between rat-free and rat-infested islands (Supplementary Tables S1 and S2, Supplementary Figs. S1 and S2).

## Discussion

Contrary to our hypothesis, nutrient subsidies from seabirds did not uniformly boost demographic rates of parrotfish on adjacent coral reefs. Instead, female parrotfish around rat-free islands with abundant seabird populations exhibited increased growth and size-at-age, but reduced reproductive investment for a given size, than parrotfish around rat-infested islands with few seabirds. Combined, these results suggest that parrotfish around rat-free versus rat-infested islands exhibit different life-history strategies. Differences in densities of breeding seabirds on these islands are likely the ultimate driver of these demographic differences, with proximate drivers including enhanced quality of food resources and/or enhanced piscivore populations, and thus increased size-selective mortality, around rat-free islands. Although these altered life-history characteristics did not correspond with observable differences in population-level biomass of *C. sordidus*, both mean size and overall size distribution were shifted toward larger individuals around rat-free islands. Furthermore, the faster growth rates and larger sizes can drive other demographic changes (e.g., lower mortality rates^55^), and increase the functional importance of parrotfish (e.g., higher bioerosion rates^21–25^). More broadly, these results have implications for understanding the extent and scales at which cross-ecosystem nutrient subsidies provide benefits.

### Drivers of demographic trade-offs

Our results provide evidence for a trade-off between investing in early growth versus reproduction, with parrotfish around rat-free islands reaching larger sizes more quickly, but at the cost of limiting their size-based reproductive investment. By contrast, parrotfish around rat-infested islands exhibited the opposite pattern of slower growth rates and reduced maximum size- and age-at-sex change, but enhanced GSI for a given size. Importantly, the magnitude of these demographic differences was similar to those observed for coral-reef fishes across broad gradients in myriad biotic and environmental variables, including latitude, temperature, fishing pressure, wave exposure, resource availability, and predator density^2,4,5,35,56–60^. Such differences often occur among study sites ranging from tens to thousands of kilometres apart, whereas our study focused on nearby islands within atolls, suggesting strong local drivers of demographic variation in these parrotfish.

Where abundant, seabirds have large effects that can transfer throughout entire food webs in both terrestrial and nearshore marine systems. Their allochotonous nutrient inputs drive bottom-up changes in the demographic rates, abundance, biomass, and community structure of primary producers, primary consumers, and higher-order predators^1,10,17,61–63^. These large documented effects of seabird-derived nutrients, combined with the large difference in seabird populations between rat-free versus rat-infested islands in our study, suggest that they are the primary drivers of local demographic variation in parrotfish observed here. Indeed, a range of environmental drivers were found not to differ between the two island treatment types. Seabirds are likely driving differences in parrotfish demographics via two non-mutually exclusive pathways—(1) seabird derived-nutrients have direct bottom-up effects on parrotfish via increased food quality, and (2) seabird-derived nutrients drive larger piscivore populations, which in turn exert top-down effects on parrotfish. We explore each of these pathways in detail below.

The enhanced growth rates of *C. sordidus* around islands with seabirds is consistent with theoretical predictions and empirical results from the bottom-up effects of nutrient subsidies on resource quality and/or quantity^3,10,11,17^. Because parrotfish feed on microscopic autotrophs contained within the reef matrix and found on various substrates including tufted cyanobacteria, turf algae, crustose coralline algae, dead coral, eroded reef substrate, and sponges^19,20^, it was not feasible to directly quantify the availability or quality of their food in this study. However, live coral cover, which is negatively correlated with food availability for parrotfish^20,42^, was similar between rat-free and rat-infested islands^40^, suggesting that resource *quantity* was also comparable between island types. By contrast, several lines of evidence suggest that enhanced food *quality* is at least partially driving the observed increased growth. Nearshore reefs around rat-free islands receive large inputs of nitrogen and phosphorous derived from seabird guano^10,15,17^, both of which are typically limiting nutrients for herbivore growth^64^. The elevated nitrogen content in *C. sordidus* around rat-free islands suggests that they are assimilating these extra nutrients. Similarly, marine algae, sponges, corals, zooplankton, invertebrates, and damselfish assimilate seabird-derived nitrogen around islands with abundant seabirds^17,45,46,65,66^, and these excess nutrients have been linked to enhanced growth rates in corals and herbivorous damselfish^17,46^. Indeed, the relative magnitude of elevated growth of *C. sordidus* is similar to that of the damselfish *Plectroglyphidodon lacrymatus* from these reefs^17^, suggesting that effects pervade across taxa of differing body sizes and highlighting just how influential seabird-derived nutrient subsidies can be on coral reefs.

In light of the observed differences in nitrogen content and growth rates of parrotfish, it was somewhat surprising that there was no difference in δ^15^N. Elevated δ^15^N values are indicative of seabird-derived nitrogen in nearshore tropical marine food chains^17,45,46^, and are typically correlated with nitrogen content^45^. One explanation for the lack of δ^15^N differences in *C. sordidus* is that their preferred diet of nitrogen-fixing cyanobacteria have depleted δ^15^N values relative to other food sources, resulting in lower δ^15^N signatures in parrotfish compared to other herbivores^19,67^. Despite having overall lower δ^15^N, some types of cyanobacteria have still been shown to incorporate nitrogen from seabirds, although they have high standard deviations around these δ^15^N values^68^, which may make detection of seabird effects more difficult in cyanobacteria-feeding organisms. A more likely explanation is that seabird-derived nutrients facilitate nitrogen fixation by cyanobacteria by supplying phosphorous, as has been shown for terrestrial run-off and groundwater^69^. Such a pathway is consistent with the observed elevated nitrogen, but not δ^15^N, in *C. sordidus* feeding on cyanobacteria. The supply of phosphorous from seabird guano may also affect demographic rates of parrotfish more directly, as growth in a number of fish species is phosphorous-limited^70^. Thus, even without elevated δ^15^N values, there is a probable link between seabird-derived nutrients and food resources, nitrogen content, and demographic rates of parrotfish.

While the enhanced growth rates are consistent with bottom-up effects of nutrient subsidies, the lower size-related reproductive investment in the presence of high resource quality was counter to our expectations^71^. We found no previous examples of consumer-derived nutrient subsidies resulting in reduced fecundity or reproductive investment, although few studies have tested for this relationship^1,3^. More broadly, lab and field experiments using a range of taxa have shown that when consumers are nutrient-limited they prioritize resource allocation to maintenance or storage rather than reproduction^13^. A key question then is why did *C. sordidus* around islands with abundant seabird populations maximize early growth above reproductive investment? One likely explanation is that piscivore biomass was higher around islands with abundant seabirds^17,40^, due to both higher densities and larger sizes of predators near rat-free islands . Because fish predators are often gape-limited, faster growth rates can increase survival of prey fish by reducing the time they are susceptible to size-selective predation^25,72^. Indeed, variation in the demographic rates and size structures of lower-trophic level fishes has repeatedly been linked to piscivore population size across a gradient of fishing pressure in the Line and Hawaiian Islands^58,60,73,74^. Thus, in the presence of high predation intensity around rat-free islands, it may be more advantageous for *C. sordidus* to devote their energy to increased growth at the cost of reduced reproduction, at least while in their initial phase. In addition to these cost differences in energetic trade-offs around rat-free versus rat-infested islands, higher size-selective mortality rates for slow growers in the presence of abundant piscivores would similarly result in more fast growing individuals around rat-free islands. In other words, in addition to nutrient subsidies having a direct bottom-up effect on the demographic rates of parrotfish, seabird-derived nutrients also extend up the food web to boost the density and biomass of piscivores^17^, which in turn exert increased top-down control on parrotfish.

By contrast, it is unlikely intraspecific competition was a major driver of the observed demographic differences, because we found no difference in the population density of *C. sordidus* around rat-free versus rat-infested islands. Furthermore, although total biomass of parrotfish (Supplementary Fig. S6) and herbivores^17,40^ is higher around rat-free islands, this is unlikely to lead to more intense interspecific competition due to fine-scale niche separation among parrotfishes^19^.

Finally, related metrics that we were unable to measure may provide additional explanations for the observed growth-reproduction trade-offs. In addition to allocating energy to growth and reproduction, animals also devote energy to maintenance and storage^14^. These parrotfish were collected several years following a major coral bleaching event, a time when they had access to abundant resources^75^. Indeed, parrotfish exhibited increased growth during this time in a pan-tropical study, as declines in coral cover lead to increases in food resources for parrotfish^19,75^. Although there was no difference in coral cover between rat-free and rat-infested islands^40^, the overall low coral cover during these years likely meant that food availability was not limiting around islands with either rat status. Presumably, however, when resources are scarce seabird-derived nutrients may be even more important, possibly leading to pronounced variation in energy storage or condition. Determining how nutrient subsidies influence multiple demographic rates at other times and in other species is an important area for future research, and may further explain the patterns in energy allocation observed here.

### Implications of demographic differences

On coral reefs, demographic traits are linked to higher-order processes in part due to non-linear scaling of many traits with body size. Notably, the enhanced growth rates and larger sizes of *C. sordidus* could influence ecosystem function. As an excavating herbivore, *C. sordidus* plays a key role in bioerosion and sediment production^22,33^ with larger individuals contributing disproportionately more to these functional roles than smaller individuals^22–24^. Based on our re-paramaterized VBGF results, combined with published allometric scaling relationships for *C. sordidus*^23^, a 4-year old individual near a rat-free island will have an annual grazing rate of 46.5 m^2^ compared to just 37.3 m^2^ around a rat-infested island, and an annual bioerosion rate of 14.1 kg versus 11.3 kg (95% HPDI grazing rat-free = 42.1 to 51.3, rat-infested = 34.7 to 40.0; bioerosion rat-free = 12.8 to 15.6, rat-infested = 10.6 to 12.2).

Parrotfish, including *C. sordidus*, are also important components of subsistence and commercial harvests in many regions. Fisheries productivity is strongly influenced by growth trajectories and mortality rates^76^. Therefore, the increased growth rates of parrotfish from seabird-derived subsidies could enhance fishery productivity. Because a higher proportion of older age and size classes were observed at rat-free sites, which is strongly indicative of lower mortality rates^55^, there may be additional boosts in productivity. Importantly, standing biomass is often decoupled from fish and fishery productivity on tropical reefs^76^, so this boost in productivity can occur despite similar biomass of *C. sordidus* around rat-free and rat-infested islands. Thus, even in the absence of a consistent numeric response, seabirds can influence population and community dynamics, as well as ecosystem functioning.

While growth rates and sizes of parrotfish can be tightly linked to local ecosystem function, reproductive characteristics are linked to regional dynamics via larval dispersal. Although individual reproductive investment was higher for female parrotfish around rat-free islands, several other factors influence reproductive output and success. For example, differences in size-at-sex change can behaviourally influence reproductive success beyond individual fecundity^56^, such that a larger size-at-sex change can result in greater lifetime reproductive output despite lower size-related reproductive investment. We found greater maximum sizes and ages of female parrotfish around rat-free islands, which is consistent with the typical association between larger size-at-sex change with faster growth and larger mean size, especially when coupled with similar population densities^56^. These differences in age- and size-at-sex change between rat-free and rat-infested islands may offset the observed differences in relative reproductive investment. Furthermore, not only do larger, older females produce more eggs, but they also produce larger and higher quality eggs, and have earlier and longer spawning seasons than smaller females^77^. Increases in reproductive energy also scale allometrically with female body size for the majority of fish species, including for *C. sordidus*^78^. Thus, the observed differences in body size and age between rat-free and rat-infested islands may further influence reproductive output and success beyond GSI.

Although we could not directly measure whether population-level reproductive output differs between rat-free and rat-infested islands, conservative calculations suggest that there was no difference in total, instantaneous, reproductive potential (Supplementary Fig. S7). In other words, although individuals around rat-infested islands had higher reproductive investment for a given size, this did not result in higher population-level reproductive output. This study is only the first step in quantifying the effects of seabird nutrient subsidies on coral-reef fish reproduction, and future work detailing additional factors that influence reproductive dynamics, including sex ratios, egg quality, fecundity of males and females, and larval dispersal patterns, will clarify the regional implications of nutrient subsidies for coral-reef fish populations.

### Management implications

Many island nations are now prioritizing rat eradication as a relatively simple, low-cost management action with potential widespread benefits, including restoring seabird populations and their associated nutrient subsidies^79,80^. These results help clarify some possible outcomes of these management actions. Here, we add to growing evidence that rat eradication may benefit not only terrestrial systems, but also nearshore coral reefs^17,18,40,46,81^, as measured by higher growth rates of functionally important parrotfish near rat-free islands. However, we did not find any direct benefits of seabird-derived nutrients for reproductive investment of individuals, and there was no difference in population-level instantaneous reproductive potential between rat-free and rat-infested islands. Although there may still be lifetime reproductive benefits to fish around rat-free islands due to lower mortality rates and/or larger size-at-sex change, we currently have no evidence that increased larval export to other reefs is an additional benefit of local eradication programs. Therefore, the benefits of seabird-derived nutrient subsidies are likely spatially-restricted, meaning that managers should seek to de-rat entire archipelagos to provide the most benefits for coral reefs.

## Supplementary Information

Supplementary Information 1.

## Data Availability

All data and code are publicly available on GitHub (github.com/cbenkwitt/nutrients-fish-demography).
