# Supplementary material for: Natural nutrient subsidies alter demographic rates in a functionally important coral-reef fish
Source: Sci Rep. 2021 Jun 15;11:12575. doi: 10.1038/s41598-021-91884-y (PMC8206227; doi:10.1038/s41598-021-91884-y)
Supplement: Supplementary file 1 — Supplementary Information 1. [file 41598_2021_91884_MOESM1_ESM.pdf]

Supplementary Information for "Natural nutrient subsidies alter demographic rates in a functionally important coral-reef fish"

Cassandra E Benkwitt, Brett M Taylor, Mark G Meekan, Nicholas AJ Graham

Contents:

Supplementary Methods: Full specifications for statistical models

Supplementary Table S1: Characteristics of islands used in this study

Supplementary Table S2: Model estimates of differences in island, reef, and environmental characteristics between rat-free versus rat-infested study islands.

Supplementary Figure S1: Map of study sites and sample sizes

Supplementary Figure S2: Island, reef, and environmental characteristics of rat-free versus rat-infested study islands

Supplementary Figure S3: Difference in size-at-age of female parrotfish (Chlorurus sordidus) around rat-free versus rat-infested islands

Supplementary Figure S4: $\delta^{15}N$ in the tissues of female parrotfish (C. sordidus) around rat-free versus rat-infested islands

Supplementary Figure S5: Piscivore density and size distribution around rat-free versus rat-infested islands

Supplementary Figure S6: Density, biomass, and size frequency distribution of parrotfish (all species combined) around rat-free versus rat-infested islands

Supplementary Figure S7: Estimated instantaneous total gonad weight of female parrotfish (C. sordidus only) around rat-free versus rat-infested islands

Supplementary Methods: Full specifications for statistical models

Percent nitrogen (%N) and ratio of isotopic nitrogen ($\delta^{15}N$) were modelled following student t-distributions as:

$$\%N_{ij} \sim \text{Student}(\nu, \mu_i, \sigma)$$
$$\mu_i = \alpha_{atoll,i} + \beta_1 rat_i$$
$$\alpha_{atoll} \sim \text{Normal}(\alpha, \sigma_a)$$
$$\alpha \sim \text{Normal}(14, 10)$$
$$\beta_1 \sim \text{Normal}(0,10)$$
$$\sigma, \sigma_a \sim \text{Student}(3,0,10)$$
$$\nu \sim \text{Gamma}(2, 0.1)$$

$$\delta^{15}N_{ij} \sim \text{Student}(\nu, \mu_i, \sigma)$$
$$\mu_i = \alpha_{atoll,i} + \beta_1 rat_i$$
$$\alpha_{atoll} \sim \text{Normal}(\alpha, \sigma_a)$$
$$\alpha \sim \text{Normal}(10, 10)$$
$$\beta_1 \sim \text{Normal}(0,10)$$
$$\sigma, \sigma_a \sim \text{Student}(3,0,10)$$
$$\nu \sim \text{Gamma}(2, 0.1)$$

where "rat" indicates whether the fish was caught near an island that is rat-infested (with few seabirds) or rat-free (with abundant seabirds) and "atoll" indicates one of three atolls within which each island was located (Supplemental Material Figure 1). The priors for α were weakly informative based on the mean and range of %N and $\delta^{15}N$ values from previous studies of similar species and/or locations (Graham et al. 2018, Plass-Johnson et al. 2013). The prior for β_1 was weakly informative with a mean around zero, thus providing no indication about the direction of any potential rat versus seabird effect. Default priors from the package brms were used for σ and ν (Bürkner 2017, 2018).

Gonadosomatic index (GSI) was modelled following a normal distribution as:

$$GSI_{ij} \sim \text{Normal}(\mu_i, \sigma)$$
$$\mu_i = \alpha_{atoll,i} + \beta_1 rat_i + \beta_2 length_i$$
$$\alpha_{atoll} \sim \text{Normal}(\alpha, \sigma_a)$$
$$\alpha \sim \text{Normal}(1, 1)$$
$$\beta_1, \beta_2 \sim \text{Normal}(0,1)$$
$$\sigma, \sigma_a \sim \text{Student}(3,0,10)$$

where length was mean-centered as per the specifications for continuous predictors in the brms package. The prior for α was weakly informative based on the observed range of GSI. The priors for β_1 and β_2 were weakly informative with means around zero, thus providing no indication about the direction of any potential rat versus seabird effect or effect of length on GSI. The prior for σ was specified as in the models for nitrogen. We compared the above additive-only model to a model containing an interaction term (rat x length) using leave-one-out cross-validation. There was no improvement in model fit when an interaction term was included, and parameter estimates were nearly identical regardless of model choice, so the simpler additive-only model was used. We also compared the fits of the above linear model to a non-linear power model, but again the linear model provided a better fit so was used for all analyses. Finally, we compared the fits and estimates of the above model with a model including collection day as an additional explanatory variable. Again, we found no improvement in model fit with the inclusion of day, so we report results from the simpler model in the main text.

We repeated all of the GSI analyses with age as an explanatory variable instead of length, and again found that the linear, additive model without collection day was the best fit. In all models, the parameter estimates and HPDI's for the difference in GSI between rat-free and rat-infested islands were nearly identical, further demonstrating the robustness of this effect (length model: estimate = -0.25, 95% HPDI = -0.45 to -0.07; length model with collection day: estimate = -0.27; 95% HPDI = -0.48 to -0.08; age model: estimate = -0.31, 95% HPDI = -0.49 to -0.12; age model with collection day: estimate = -0.32, 95% HPDI = -0.52 to -0.14).

The VBGF model for size (L) at age (t) was specified as:

$$\ln(L_{ij}) \sim \text{Normal}(\mu_i, \sigma)$$

$\mu_i = \ln(L_\infty - (L_\infty - L_0)e^{-(k \ \ +k_b)t})$

L_∞ ~ Uniform(min(L), max(L) x 2)

L_0 ~ Uniform(0, min(L))

k ~ Uniform(0,2)

k_b ~ Normal(0,1)

σ ~ Student(3,0,10)

Priors for L_∞, L_0, k, and k_b were set following Graham et al. (2018), which used the same VBGF specification to compare damselfish growth between rat-infested and rat-free islands. Default priors were used for σ. A log-log specification was used to improve model convergence. We compared the above model where *k* was allowed to vary by rat status to a model where both L_∞ and *k* were allowed to vary by rat status. The estimated growth curves and difference in *k* between rat-free and rat-infested models were similar in the two models (*k*-only model: estimated *k* = 0.27, 95% HPDI = 0.16 to 0.41; estimated k_b = 0.10, 95% HPDI = 0.02 to 0.20; both *k* and L_∞ model: *k* = 0.26, 95% HPDI = 0.07 to 0.50; k_b = 0.09, 95% HPDI = -0.17 to 0.31). There was also no evidence that L_∞ differed between rat-free and rat-infested islands even when it was allowed to vary (estimated difference in L_∞ = 0.06, 95% HPDI = -7.51 to 4.83). Furthermore, the model fit was best when only *k* was allowed to vary by rat status, compared to the model in which both L_∞ and *k* were allowed to vary, as well as a null model in which neither were allowed to vary.

To test for a correlation between GSI and growth rate within individual parrotfish, we ran an additional VBGF model in which *k* was allowed to vary by individual rather than by rat status. We extracted these individual *k* estimates and conducted a Bayesian correlation analysis between measured GSI and model-estimated *k* using the R package *correlation* (Makowski et al. 2020).

The re-parameterized VBGF model for size (L) at age (t) was specified as:

$\ln(L_{ij})$ ~ Normal(μ_i, σ)

$$\mu_i = \ln(L_{phi} + (L_{psi} - L_{phi})(1 - r^{\frac{2(t-phi)}{psi-phi}})/(1 - r^2))$$

where $r = (L_{phi} - L_{chi})/(L_{chi} - L_{psi})$ and $phi = 2$, $psi = 6$, and $chi = 4$

$L_{phi} \sim$ Uniform(min(L), max(L) x 2)

$L_{psi} \sim$ Uniform(min(L), max(L) x 2)

$L_{chi} \sim$ Uniform(min(L), max(L) x 2)

$L_{phi\ b} \sim$ Normal(0,10)

$L_{psi\ b} \sim$ Normal(0,10)

$L_{chi\ b} \sim$ Normal(0,10)

$\sigma \sim$ Student(3,0,10)

Each length-at-age (L_{phi}, L_{psi}, L_{chi}) had a corresponding offset to allow it to vary by rat status ($L_{phi\ b}$, $L_{psi\ b}$, $L_{chi\ b}$), similar to that for k in the VBGF model above. A log-log specification was used to improve model convergence. Priors for length-at-ages were constrained between the minimum and twice the maximum of observed lengths. Default priors were used for σ.

Maximum length (Lmax) and age (Tmax) of female *C. sordidus* were modelled following normal distributions as:

$Lmax_{ij} \sim$ Normal (μ_i, σ)

$\mu_i = \alpha_{atoll,i} + \beta_1 rat_i$

$\alpha_{atoll} \sim$ Normal(α, σ_a)

α ~ Normal(23, 5)

$\beta_1 \sim$ Normal(0,10)

σ, $\sigma_a \sim$ Student(3,0,10)

$Tmax_{ij} \sim$ Normal(μ_i, σ)

$\mu_i = \alpha_{atoll,i} + \beta_1 rat_i$

$\alpha_{atoll} \sim$ Normal(α, σ_a)

α ~ Normal(6, 2)

$\beta_1 \sim$ Normal(0,10)

σ, $\sigma_a \sim$ Student(3,0,10)

The priors for α were weakly informative based on the observed range of maximum age and length. The priors for β_1 were weakly informative with means around zero, thus providing no indication about the direction of any potential rat versus seabird effect or effect. Default priors were used for σ.

Population density (D) and biomass (B) of *C. sordidus* from visual surveys were modelled as:

$D_{ij} \sim \text{HurdleGamma}(\mu_i, \text{shape}, \text{hu})$

$\ln(\mu_i) = \alpha_{atoll,i} + \beta_1 rat_i$

$\alpha_{atoll} \sim \text{Normal}(\alpha, \sigma)$

$\alpha \sim \text{Normal}(0, 1)$

$\beta_1 \sim \text{Normal}(0,1)$

$\sigma, \sigma_a \sim \text{Student}(3,0,10)$

$\text{shape} \sim \text{Gamma}(0.01, 0.01)$

$\text{hu} \sim \text{Beta}(1,1)$

$B_{ij} \sim \text{HurdleGamma}(\mu_i, \text{shape}, \text{hu})$

$\ln(\mu_i) = \alpha_{atoll,i} + \beta_1 rat_i$

$\alpha_{atoll} \sim \text{Normal}(\alpha, \sigma)$

$\alpha \sim \text{Normal}(0, 1)$

$\beta_1 \sim \text{Normal}(0,1)$

$\sigma, \sigma_a \sim \text{Student}(3,0,10)$

$\text{shape} \sim \text{Gamma}(0.01, 0.01)$

$\text{hu} \sim \text{Beta}(1,1)$

Hurdle gamma distributions with log links were used because the data consisted of non-negative, continuous values and were zero-inflated. Weakly informative priors were used for α and β_1, and default priors were used for σ, hu, and shape parameters.

The frequency distribution of *C. sordidus* size (length, L) around rat-free versus rat-infested islands was modelled as:

$L_{ij} \sim \text{ExGaussian}(\mu_i, \text{beta}, \sigma)$

$\mu_i = \alpha_{atoll,i} + \beta_1 rat_i$

$\alpha_{atoll} \sim \text{Normal}(\alpha, \sigma_a)$

$\alpha \sim \text{Normal}(15, 1)$

$\sigma_a \sim \text{Student}(3,0,10)$

$\beta_1 \sim \text{Normal}(0,10)$

$\text{beta} \sim \alpha_i + \beta_2 rat_i$

$\alpha_i \sim \text{Lognormal}(0,1)$

$\beta_2 \sim \text{Normal}(0,1)$

$\sigma \sim \text{Lognormal}(0,1)$

An exponentially-modified gaussian model was used because the data were right-skewed, and using this distribution allowed explicit modelling of both the mean (μ) and the right-skewness (beta). Thus, both μ and beta were allowed to vary as a function of rat

presences. In addition, model comparisons and posterior predictive checks indicated that an exponentially-modified gaussian distribution provided the best fit. Weakly informative priors were used for α's and β's, and default priors were used for σ.

Finally, to determine whether there were differences in other island, reef, and environmental characteristics between rat-free and rat-infested islands, we modelled seabird density, island size, wave energy, net primary productivity, coral cover, structural complexity, and piscivore biomass, density, and size as a function of rat status with a random intercept for atoll. As for the other models, we specified weakly informative priors for α's and β's, and default priors for σ. For variables that had data available from all islands (seabird density, island size, wave energy, and net primary productivity), we ran analyse with all islands (n = 14), as well as with only islands where parrotfish were collected (n = 8), and only islands where visual surveys were conducted (n = 11). In all cases, results were similar regardless of which islands were used in the analysis (Supplementary Tables S1 and S2, Supplementary Fig. S2). All data and code are publicly available on GitHub (github.com/cbenkwitt/nutrients-fish-demography).

References

Bürkner PC. 2017 brms: An R Package for Bayesian Multilevel Models Using Stan. *Journal of Statistical Software* **80**, 1–28.

Bürkner PC. 2018 Advanced Bayesian Multilevel Modeling with the R Package brms. *The R Journal* **10**, 395–411.

Graham NAJ, Wilson SK, Carr P, Hoey AS, Jennings S, MacNeil MA. 2018 Seabirds enhance coral reef productivity and functioning in the absence of invasive rats. *Nature* **559**, 250–253. (doi:10.1038/s41586-018-0202-3)

Makowski D, Ben-Shachar MS, Patil I, Lüdecke D. 2019 Methods and algorithms for correlation analysis in R. *Journal of Open Source Software* **5**, 2306. (doi: 10.21105/joss.02306)

Plass-Johnson JG, McQuaid CD, Hill JM. 2013 Stable isotope analysis indicates a lack of inter- and intra-specific dietary redundancy among ecologically important coral reef fishes. *Coral Reefs* **32**, 429–440. (doi:10.1007/s00338-012-0988-7)

Supplementary Table S1: Characteristics of islands used in this study

rat status	island	atoll	latitude (degrees south)	longitude (degrees east)	collections	surveys	seabird density (breeding pairs/ha)	island area (ha)	net primary productivity (mg C/(m^2 day))	wave energy (kW/m)	sea surface temperature (°C)	piscivore biomass (kg/ha)	coral cover (%)	structural complexity (0-5 scale)
rat-free	Middle Brother	GCB	-6.15204	71.51846	X	X	4034.38	8	864.92	29.51	28.85	443.40	10.42	2.88
rat-free	South Brother	GCB	-6.16904	71.5437		X	975.30	23	886.42	29.73	28.89	12.45	10.83	1.00
rat-free	Grande Ile Coquillage	PB	-5.37241	71.96729	X	X	1447.32	28	765.96	0.48	29.15	37.30	4.17	1.63
rat-free	Petite Ile Coquillage	PB	-5.3403	71.97078	X		1054.05	21	765.96	0.48	29.15			
rat-free	Ile Longue	PB	-5.27221	71.86643		X	1951.29	25.5	724.83	22.86	29.11	57.30	0.83	2.25
rat-free	Ile de la Passe	SAL	-5.30566	72.25172	X	X	19.15	26	700.58	0.31	29.15	49.48	56.38	3.13
rat-infested	Eagle Island	GCB	-6.18665	71.33818	X	X	0.33	243.5	843.61	30.29	28.86	8.73	15.84	1.50
rat-infested	Diamante	PB	-5.25665	71.76511	X		0.63	91	698.90	0.38	29.26			
rat-infested	Ile Gabrielle	PB	-5.42424	71.75498	X		0.94	16	783.83	0.24	29.15			
rat-infested	Ile Anglaise	PB	-5.43892	71.75688		X	3.33	12	783.83	0.24	29.15	63.58	13.75	2.13
rat-infested	Ile Poule	PB	-5.41052	71.75508		X	0.15	108	787.89	0.41	29.09	0.95	9.58	1.88
rat-infested	Grande Ile Mapou	PB	-5.2663	71.75596		X	2.31	19.5	698.90	0.38	29.26	20.15	5.00	1.88
rat-infested	Ile Anglaise	SAL	-5.32822	72.22462	X	X	0.61	75.5	753.31	0.23	29.20	44.78	43.18	3.25
rat-infested	Ile Fouquet	SAL	-5.3431	72.26185		X	2.76	39.5	718.04	24.71	29.15	0.00	28.79	3.00
			all islands			*mean rat-free islands (SE)*	*1580.2 (226.8)*	*21.9 (1.2)*	*784.8 (12.5)*	*13.9 (2.5)*	*29.0 (0.02)*	*120.0 (36.3)*	*16.5 (4.5)*	*2.2 (0.2)*
						mean rat-infested islands (SE)	*1.4 (0.2)*	*75.6 (9.6)*	*758.5 (6.4)*	*7.1 (1.6)*	*29.1 (0.02)*	*23.0 (4.3)*	*19.4 (2.4)*	*2.3 (0.1)*
			survey islands only			*mean rat-free islands (SE)*	1638.7 (426.7)	20.8 (2.2)	774.4 (16.9)	7.7 (3.6)	29.1 (0.04)	176.7 (77.0)	23.7 (9.5)	2.5 (0.3)
						mean rat-infested islands (SE)	0.63 (0.1)	106.5 (24.2)	769.9 (15.1)	7.8 (3.8)	29.1 (0.04)	26.8 (12.7)	29.5 (9.7)	2.4 (0.6)
			collection islands only			*mean rat-free islands (SE)*	1685.5 (298.8)	22.1 (1.6)	788.5 (16.6)	16.6 (3.0)	29.0 (0.03)	*120.0 (36.3)*	*16.5 (4.5)*	*2.2 (0.2)*
						mean rat-infested islands (SE)	1.6 (0.2)	83.0 (14.4)	764.3 (8.7)	9.4 (2.4)	29.1 (0.02)	*23.0 (4.3)*	*19.4 (2.4)*	*2.3 (0.1)*

Table S1 data sources: seabird breeding pairs from Carr et al. (2020); net primary productivity from Yeager et al. (2017) (https://shiny.sesync.org/apps/msec/); wave energy from Yeager et al. (2017) (https://shiny.sesync.org/apps/msec/) and corroborated with Perry et al. 2015; sea surface temperate from NOAA (https://coastwatch.pfeg.noaa.gov/erddap/index.html), monthly averages from 2011-2019 which spans entire age range of oldest fish captured; piscivore biomass, coral cover, and structural complexity from Benkwitt et al. (2019).

Supplementary Table S2: Model estimates of differences in island, reef, and environmental characteristics between rat-free versus rat-infested study islands.

Table S2. Median difference and 95% highest posterior density interval (HPDI) of rat-free compared to rat-infested islands from Bayesian models. Where applicable, models were run for all study islands combined, collection islands only, and survey islands only. See also Table S1 and Figure S2.

Response	Islands	Estimated difference (95% HPDI)
Island area (log ha)	All	-0.68 (-1.61 to 0.27)
	Collection only	-0.86 (-2.09 to 0.48)
	Survey only	-0.70 (-1.73 to 0.47)
Net primary productivity (mg C/(m^2 day))	All	3.77 (-38.89 to 51.79)
	Collection only	3.35 (-63.49 to 72.42)
	Survey only	-2.67 (-57.67 to 51.09)
Wave energy (kW/m)	All	3.01 (-6.51 to 12.91)
	Collection only	-0.09 (-0.76 to 0.66)
	Survey only	3.54 (-7.62 to 15.34)
Sea surface temperature (^{o}C)	All	-0.03 (-0.10 to 0.04)
	Collection only	-0.04 (-0.15 to 0.06)
	Survey only	-0.02 (-0.11 to 0.07)
Coral cover (%)	Survey only	0.55 (-5.22 to 6.27)
Structural complexity (0-5 scale)	Survey only	0.09 (-0.31 to 0.43)

Supplementary Figure S1: Map of study sites and sample sizes

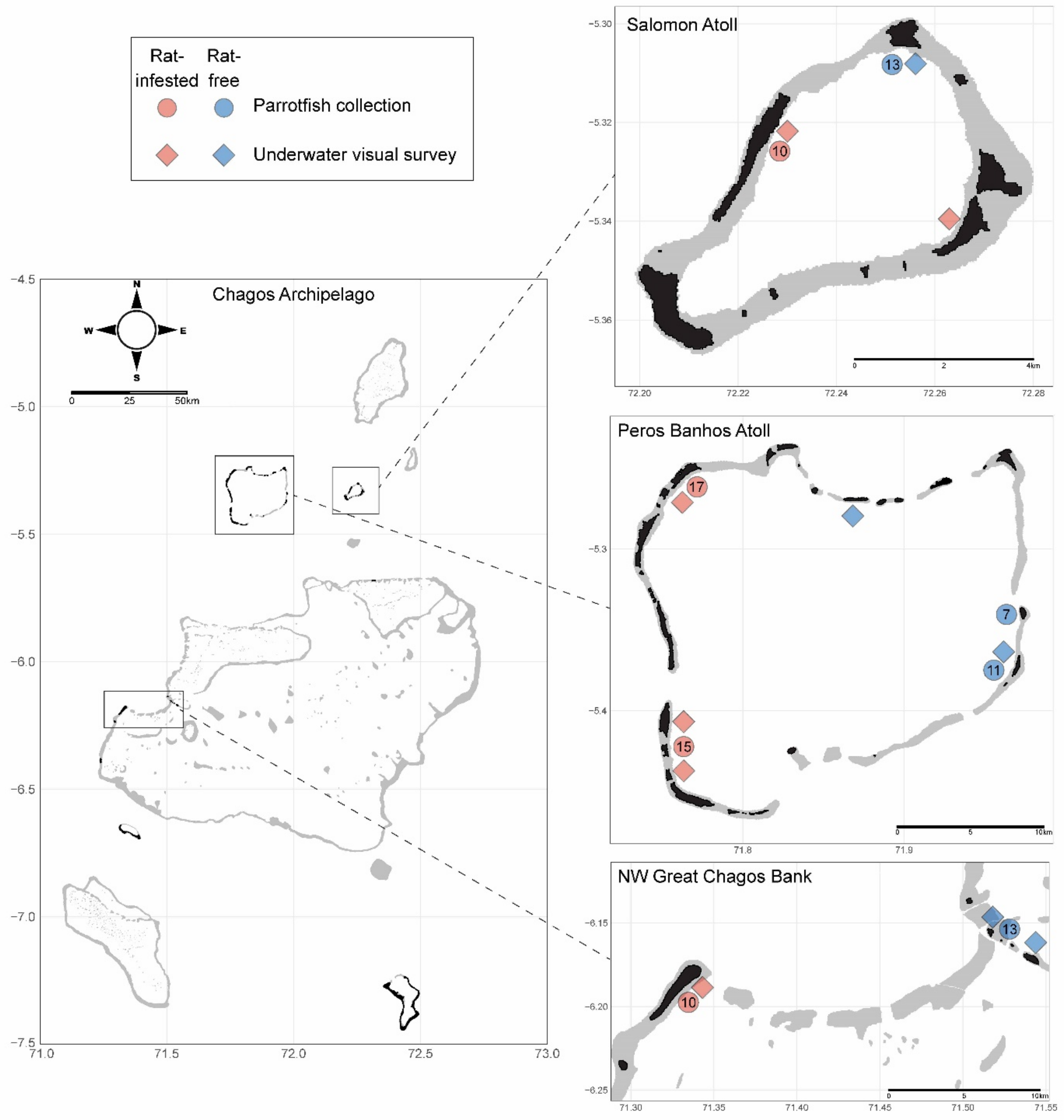

Supplementary Figure S1. Study sites within the Chagos Archipelago, Indian Ocean. All sites were located on the reef crest and shallow slope adjacent to islands that were either rat-infested (with few seabirds) or rat-free (with abundant seabirds). Locations where parrotfish (*Chlorurus sordidus*) were collected are indicated by circles, with sample size of spawning-capable females (used in all analyses) collected from each island provided in the circle. Locations where visual surveys were conducted are indicated by diamonds (n = 4 transects [150 m^2 each] per island). Map was created using R version 3.6.1.

Supplementary Figure S2: Island, reef, and environmental characteristics of rat-free versus rat-infested study islands

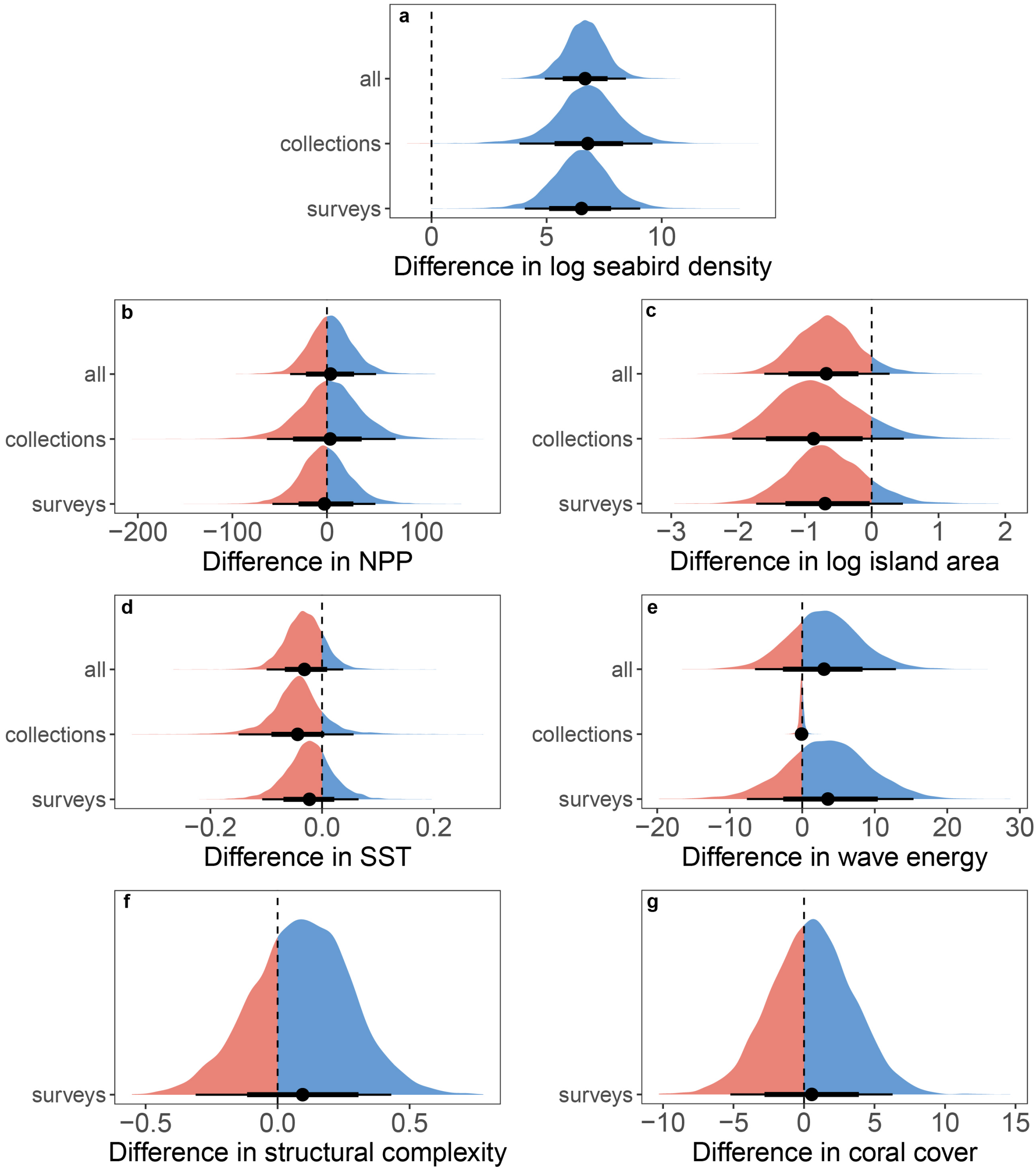

Supplementary Figure S2. Bayesian posterior densities for the difference in additional island, reef, and environmental characteristics of rat-free versus rat-infested islands. Positive values of the posterior distributions (blue fill) indicate the response is higher on or around rat-free island, negative values (red fill) indicate the response is lower on or around rat-free islands. Points represent median highest posterior density estimate and lines represent 75% and 95% highest posterior distribution intervals (HPDI). See also Figure 3, Tables S1-S2.

Supplementary Figure S3: Difference in size-at-age around rat-free versus rat-infested islands

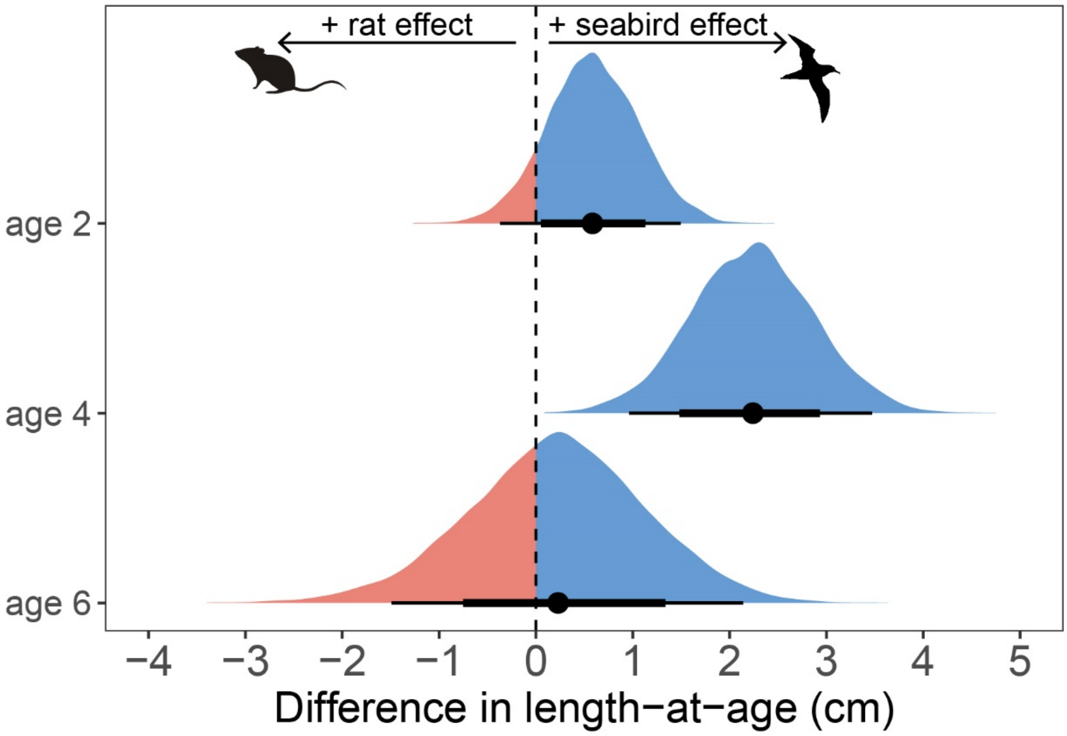

Supplementary Figure S3. Bayesian posterior densities for the effect of seabird presence on length-at-age from the reparameterized von Bertalanffy growth model. Positive values of the posterior distributions (blue fill) indicate a positive effect of seabird presence on the response, negative values (red fill) indicate a negative effect of seabirds (i.e., positive effect of invasive rat presence). Points represent median highest posterior density estimate and lines represent 75% and 95% highest posterior distribution intervals (HPDI). Rat and seabird silhouettes were obtained from phylopic.org under Public Domain Dedication 1.0 licenses.

Supplementary Figure S4: δ^{15}N in the tissues of female parrotfish (C. sordidus) around rat-free versus rat-infested islands

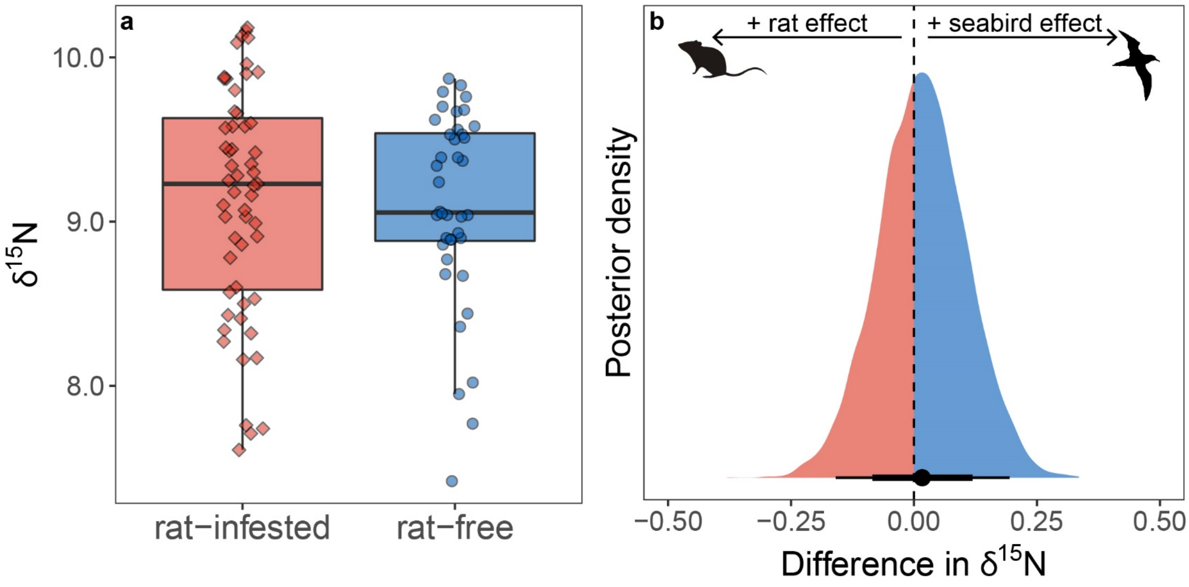

Supplementary Figure S4. δ^{15}N in the tissues of female parrotfish, *Chlorurus sordidus*, around rat-free islands with abundant seabirds (blue, circles) compared to rat-infested islands with few seabirds (red, diamonds). (a) Points represent measured values from individual *C. sordidus*. Box limits represent first and third quantiles (25% and 75% percentiles), middle line represents the median (50% percentile), and whiskers represent smallest and largest observations less than or equal to 1.5x inter-quartile range. (b) Bayesian posterior densities for the effect of seabird presence on δ^{15}N. Points represent median highest posterior density estimate and lines represent 75% and 95% highest posterior distribution intervals (HPDI). Rat and seabird silhouettes were obtained from phylopic.org under Public Domain Dedication 1.0 licenses.

Supplementary Figure S5: Difference in piscivore density and size distribution around rat-free versus rat-infested islands.

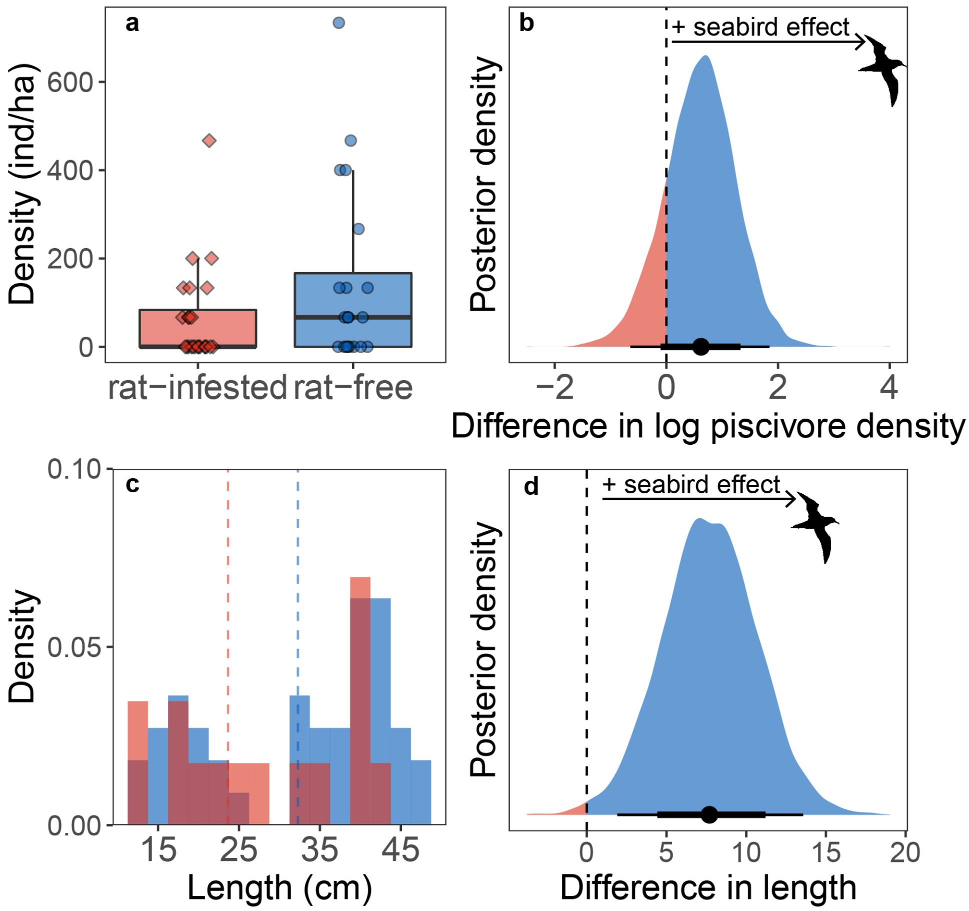

Supplementary Figure S5. Raw data and model estimates for piscivore density and size distribution around rat-free islands with abundant seabirds (blue, circles) compared to rat-infested islands with few seabirds (red, diamonds). (a) Box limits represent first and third quantiles (25% and 75% percentiles), middle line represents the median (50% percentile), whiskers represent smallest and largest observations less than or equal to 1.5x inter-quartile range, and each point represents on transect. (c) Size frequency distribution of piscivores in 2.5 cm size bins, dotted lines represent mean length. (b,d) Bayesian posterior densities for the effect of seabird presence on piscivore density and size. Positive values of the posterior distributions (blue fill) indicate a positive effect of seabird presence on the response, negative values (red fill) indicate a negative effect of seabirds (i.e., positive effect of invasive rat presence). Points represent median highest posterior density estimate and lines represent 75% and 95% highest posterior distribution intervals (HPDI). Rat and seabird silhouettes were obtained from phylopic.org under Public Domain Dedication 1.0 licenses.

Supplementary Figure S6: Biomass, density, and size frequency distribution of parrotfish (all species combined) around rat-free versus rat-infested islands.

Models for biomass and density were specified in the same way as models for *C. sordidus* (Supplementary Methods), but included all species of parrotfish. The size distribution of all parrotfish was modelled following a skew normal distribution, rather than an exponentially-modified gaussian distribution as used for *C. sordidus*, because the distribution was skewed, but not always right-skewed as for *C. sordidus*.

$L_{ij} \sim \text{SkewNormal}(\mu_i, \text{alpha}, \sigma)$

$\mu_i = \alpha_{atoll,i} + \beta_1 rat_i$

$\alpha_{atoll} \sim \text{Normal}(\alpha, \sigma_a)$

$\alpha \sim \text{Normal}(23, 10)$

$\beta_1 \sim \text{Normal}(0,10)$

$\text{alpha} \sim \alpha_i + \beta_2 rat_i$

$\alpha_i \sim \text{Normal}(0,4)$

$\beta_2 \sim \text{Normal}(0,10)$

$\sigma, \sigma_a \sim \sim \text{Student}(3,0,10)$

Similar to the beta parameter in the exponentially-modified Gaussian distribution, the alpha parameter in the skew normal distribution indicates the skewness, and both the mean and this skewness parameter were allowed to vary by the presence of rats. As above, weakly informative priors were used for α's and β's, and default priors were used for σ.

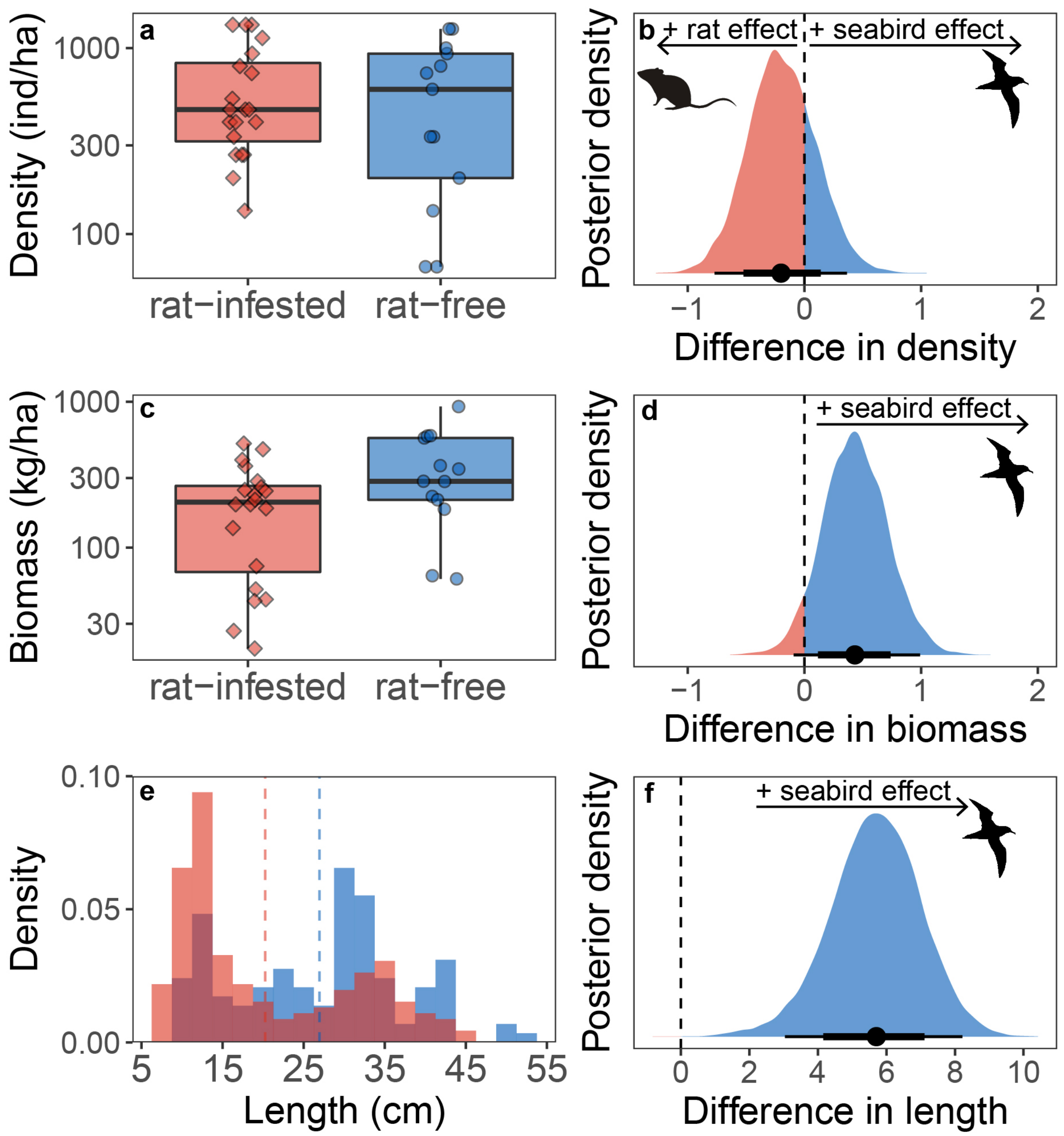

Supplementary Figure S6. Density, biomass, and size frequency distribution of all parrotfish combined around rat-free islands with abundant seabirds (blue, circles) compared to rat-infested islands with few seabirds (red, diamonds). (a,c) Box limits represent first and third quantiles (25% and 75% percentiles), middle line represents the median (50% percentile), and whiskers represent smallest and largest observations less than or equal to 1.5x inter-quartile range. Each point represents one transect. Transects on which no parrotfish were observed were excluded from the boxplots (n = 3 around rat-infested islands, n = 7 around rat-free islands) to better correspond to estimates from non-zero components of hurdle gamma models presented in (b, d). (e) Size frequency distribution of parrotfish in 2.5 cm size bins. (b,d,f) Bayesian posterior densities for the effect of seabird presence on corresponding responses in (a, c, e). Points represent median highest posterior density estimate and lines represent 75% and 95% highest posterior distribution intervals (HPDI). Rat and seabird silhouettes were obtained from phylopic.org under Public Domain Dedication 1.0 licenses.

Supplementary Figure S7: Estimated instantaneous total gonad weight of female parrotfish (C. sordidus only) around rat-free versus rat-infested islands.

We conducted back-of-the-envelope calculations to approximate instantaneous population-level gonad weight (as a proxy for potential reproductive output) of female parrotfish around rat-free versus rat-infested islands. We used published estimates of size-at-sex change for *C. sordidus*, combined with the observed sexes of *C. sordidus* at a range of lengths caught within our study area, to approximate which fish from our underwater visual census surveys were females. Therefore, an important assumption of this estimation is that size-at-sex change is similar between rat-free and rat-infested islands. Given that it is more likely that size-at-sex change is greater around rat-free islands (see *Discussion*), if anything these calculations likely underestimate total potential reproductive output around rat-free islands relative to rat-infested islands. We estimated that any *C. sordidus* between 10 and 27 cm total length was female. We obtained qualitatively similar results when re-running the analysis using different length cut-offs.

We first derived gonad weight-length relationships for *C. sordidus* around rat-free and rat-infested islands. Gonad weight (GW) was modelled as a function of length following a normal distribution as:

$$GW_{ij} \sim \text{Normal}(\mu_i, \sigma)$$
$$\mu_i = \alpha_{atoll,i} + \beta_1 rat_i + \beta_2 length_i$$
$$\alpha_{atoll} \sim \text{Normal}(\alpha, \sigma_a)$$
$$\alpha \sim \text{Normal}(2, 10)$$
$$\beta_1 \sim \text{Normal}(0,10)$$
$$\beta_2 \sim \text{Lognormal}(0,1)$$
$$\sigma, \sigma_a \sim \text{Student}(3,0,10)$$

where length was mean-centered as per the specifications for continuous predictors in the brms package. The prior for α was weakly informative based on the observed range of

gonad weights. The prior for β_2 was constrained to stay above zero because the relationship between length and gonad weight is always positive. The priors for β_1 and σ were specified as in the models for nitrogen. We compared the above additive-only model to a model containing an interaction term (rat x length) using leave-one-out cross-validation. There was no improvement in model fit when an interaction term was included, and parameter estimates were nearly identical regardless of model choice, so the simpler additive-only model was used.

We then estimated gonad weight, along with 95% highest posterior density (HPD) intervals, of each female from the population-level suveys based on its observed length. We summed these estimated gonad weights across all females observed on each transect to estimate total gonad weight. Total gonad weight was modelled as a function of rat presence, with the intercept allowed to vary by atoll, following a hurdle gamma model using a log link (similar to models for biomass and density, specified in Supplemental Material SM2). As for all other models, weakly informative priors were used for the intercept (α) and slope (β) parameters, and default priors were used for σ, hu, and shape parameters. In addition to modelling the estimated gonad weights, we re-ran the models using the upper and lower limits of the 95% HPD intervals, and obtained similar results in all cases.

Importantly, this analysis provides an approximate, instantaneous estimate of population-level gonad weight at one point in time, and therefore does not account for any potential differences in the timing and/or length of the spawning season around rat-free versus rat-infested islands. Despite the caveats, this analysis is useful in that it provides the first estimation of population-level potential relative reproductive output, around rat-free versus rat-infested islands, and serves as a comparison to the results for individual-level reproductive investment around these same islands.

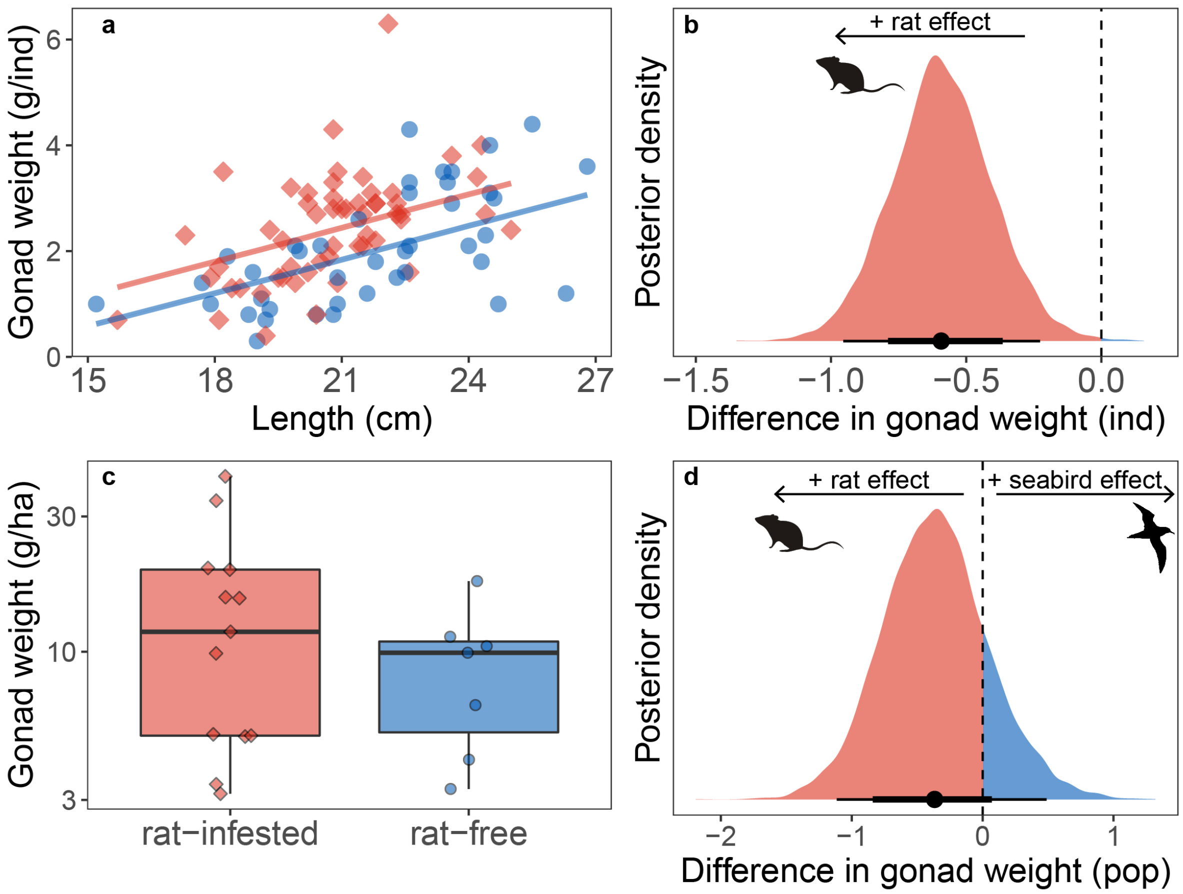

Supplementary Figure S7. Estimated gonad weight of individual *Chlorurus sordidus* and total gonad weight of all female *Chlorurus sordidus* around rat-free islands with abundant seabirds (blue, circles) compared to rat-infested islands with few seabirds (red, diamonds). (a) Relationship between individual gonad weight and length around rat-free versus rat-infested islands. Points represent measured values form individual *C. sordidus,* curves represent fitted estimates from Bayesian models. (b) Bayesian posterior densities for the effect of seabird presence on individual gonad weight. (c) Box limits represent first and third quantiles (25% and 75% percentiles), middle line represents the median (50% percentile), and whiskers represent smallest and largest observations less than or equal to 1.5x inter-quartile range. Each point represents the summed gonad weight of all female *C. sordidus* on one transect. Transects on which no female *C. sordidus* were observed were excluded from the boxplots (n = 11 around rat-infested islands, n = 13 around rat-free islands) to better correspond to estimates from non-zero components of hurdle gamma models presented in (d). (d) Bayesian posterior densities for the effect of seabird presence on summed gonad weight. (b,d) Point represents median highest posterior density estimate and lines represent 75% and 95% highest posterior distribution intervals (HPDI). Rat and seabird silhouettes were obtained from phylopic.org under Public Domain Dedication 1.0 licenses.
